# The Relative Effects of Monotherapies for Psoriatic Nails: A Network Meta‐Analysis Study

**DOI:** 10.1111/jocd.70657

**Published:** 2026-01-07

**Authors:** Aditya K. Gupta, Mary A. Bamimore, Tong Wang, Mesbah Talukder

**Affiliations:** ^1^ Mediprobe Research Inc. London Ontario Canada; ^2^ Division of Dermatology, Temerty Faculty of Medicine University of Toronto Toronto Ontario Canada; ^3^ School of Pharmacy BRAC University Dhaka Bangladesh

**Keywords:** biologics, nail psoriasis, NAPSI, network meta‐analysis

## Abstract

**Background:**

Various treatments exist for nail psoriasis (NP). We determined the relative efficacy of various monotherapies through Bayesian network meta‐analyses (NMAs).

**Methods:**

We systematically reviewed the literature to identify eligible studies which—within the patient, intervention, comparator, outcome (PICO) context—determined the impact of biologic monotherapies on NP in terms of two outcome measures, namely, (1) the 16 to 24‐week mean change in the Nail Psoriasis Severity Index (NAPSI) (i.e., outcome 1), and (2) the proportion who attained a Physician Global Assessment of fingernails (PGA‐f) of “0” or “1” (i.e., “clear” or “almost clear”) between 16 and 24 weeks (i.e., outcome 2). Our NMAs estimated the surface under the cumulative ranking curve (SUCRA) values and pairwise relative effects. We also determined relative effects of comparators that had never been compared for this condition, including “deucravacitinib 6mg daily”, “risankizumab 150 mg at weeks 0, 4, 16”, and “golimumab 2mg/kg at weeks 0, 4, then every 8 weeks”.

**Results and Conclusion:**

We identified 22 active comparators; “tofacitinib 10 mg twice daily” was ranked most efficacious in terms of 16 to 24‐week mean change in NAPSI (SUCRA = 99.71%)—while “ixekizumab 160 mg at week 0 followed by 80 mg every 4 weeks” was ranked most efficacious (SUCRA = 95.67%) for proportion attaining PGA‐f of 0 or 1 (i.e., outcome 2). Our analyses produced comparative evidence for the relative efficacy of monotherapies with various agents, including biologics and non‐biologics. Our findings would guide clinical decision‐making.

## 1 Introduction

Psoriasis is a chronic, multisystem autoimmune disorder that can involve the skin, nails, or joints [1, 2]. While the prevalence of psoriasis can vary across countries, the global prevalence is about 2% [3] and, globally, approximately 60 million individuals have psoriasis [1]. The global lifetime incidence of developing nail psoriasis (NP) among persons with plaque psoriasis is 80% to 90% [4, 5]; furthermore, NP is more prevalent in males than in females [6, 7]. Beyond physical disability [6, 7], a diagnosis of psoriasis has been highly associated with stigmatization [8], and anxiety [9].

Over the last decade, several immunomodulatory agents, such as biologics, have been used to treat NP, and their relative efficacy has been published in a couple of network meta‐analyses (NMAs) [10, 11, 12, 13]. The current study used the existing evidence base to determine the relative efficacy of monotherapy with biologics on NP using NMAs.

## 2 Materials and Methods

The entire conduct of our work was in accordance with the *Preferred Reporting Items for Systematic Reviews and Meta‐Analyses* (PRISMA) guidelines for NMAs [14]. The protocol for the current study was registered in the Open Science Framework (OSF) [15] platform (https://doi.org/10.17605/OSF.IO/34KWR).

As per the patient, intervention, comparator, outcome (PICO) framework [16] for eligible studies: our population of interest was persons with NP, and the outcomes of interest were the following: (1) mean change in Nail Psoriasis Severity Index (NAPSI) between 16 and 24 weeks from baseline and (2) proportion of patients who attained a Physician Global Assessment of “0” or “1” (i.e., “clear” or “almost clear”) in fingernails (PGA‐f) between 16 and 24 weeks from baseline. The intervention of interest was monotherapy with an immunomodulatory agent (i.e., biologics)—as well as commonly used ones (e.g., methotrexate, cyclosporine, etc.)—for NP; comparators could be placebo/vehicle or other active comparators. Furthermore, eligible studies had to be published in English.

Systematic searches were conducted in PubMed and Scopus. The Systematic Review Accelerator (SRA) [17] was used to deduplicate the searches; Rayyan [18] software was used for the two stages of screening, that is, screening of (1) titles and abstracts and (2) full texts. The searches and screenings were done independently by two authors (M.T. and M.A.B.); disagreements were resolved through discussion with a third author (A.K.G.). Following the review of full texts, data extraction commenced, and the extracted data were organized in spreadsheets.

For each of the two outcomes of interest, we conducted a Bayesian NMA with uniform priors to estimate pairwise relative effects and surface under the cumulative ranking curve (SUCRA) values. For the 16 to 24 week change in mean NAPSI, point estimate for pairwise relative effects was the mean difference (MD); the odds ratio (OR) was used for the other outcome of interest, that is, proportion of patients who attained a PGA‐f of 0 or 1 between 16 and 24 weeks. The 95% credible interval (CI) was estimated for point estimates.

We used network plots to depict the geometry of the networks; the conduct of node‐splitting analyses for consistency was dependent on the networks' geometry. For sensitivity analyses, we conducted a network meta‐regression that adjusted for variation in sex distribution ecologically (i.e., at the study level).

## 3 Results

**FIGURE 1:**
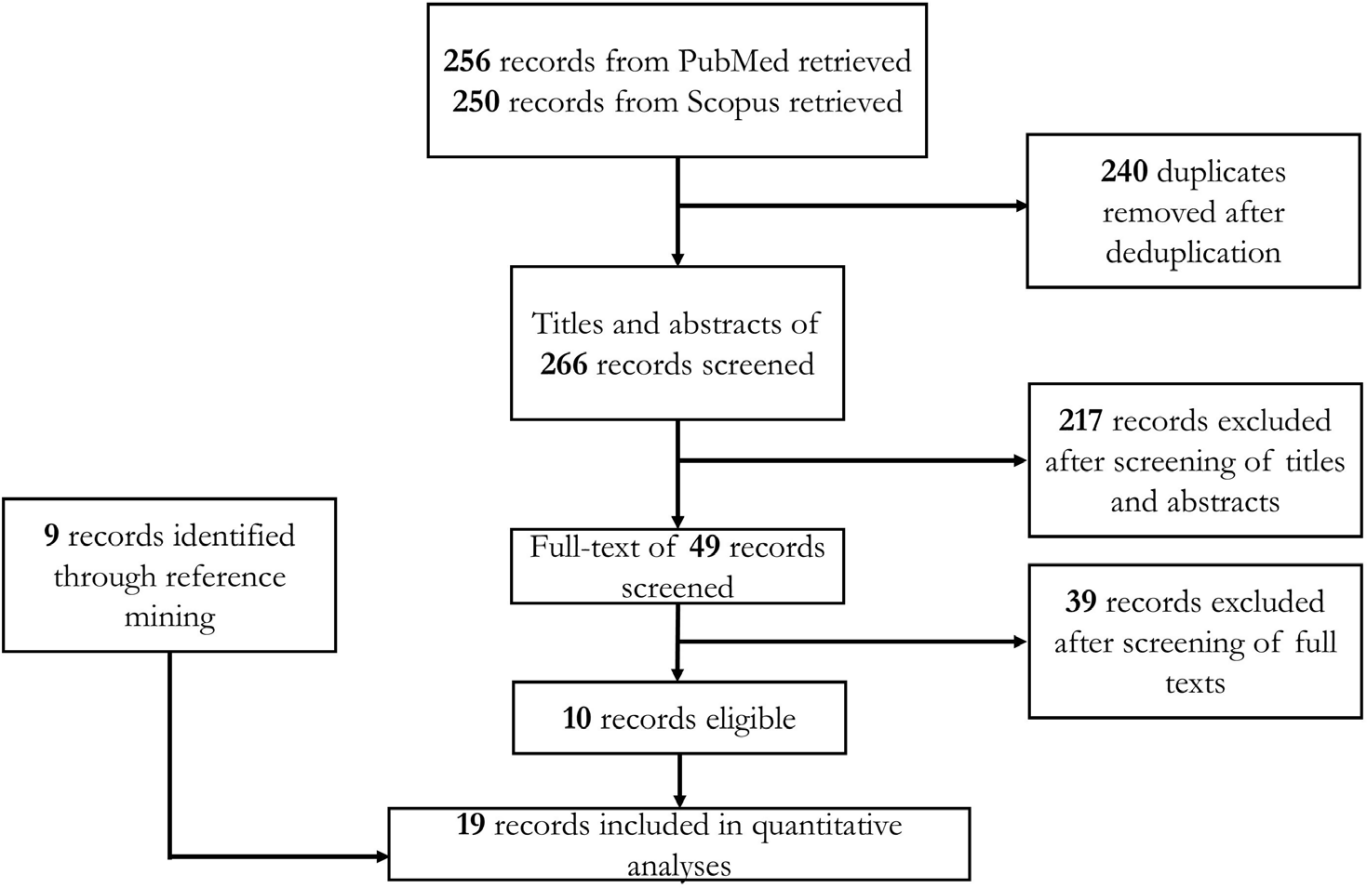
Summary of search process. This flow chart briefly depicts the systematic process entailed in our search for eligible studies.

The data used across all our analyses were obtained from a total of 19 studies [19, 20, 21, 22, 23, 24, 25, 26, 27, 28, 29, 30, 31, 32, 33, 34, 35, 36, 37] (Figure 1) whose study‐level characteristics have been summarized in Table 1. A total of 22 active comparators were identified, namely, (1) guselkumab 100 mg at weeks 0, 4, 12, 20, (2) adalimumab 40 to 80 mg at week 0; 40 mg every other week, (3) deucravacitinib 6 mg daily, (4) ixekizumab 160 mg at week 0; 80 mg every 4 weeks, (5) risankizumab 150 mg at weeks 0, 4, 16, (6) etanercept 50 mg twice a week for 12 weeks; 25 mg twice a week for 12 weeks, (7) infliximab 5 mg/kg at weeks 0, 2, 6; every 8 weeks till week 24, (8) etanercept 50 mg once a week for 24 weeks, (9) ixekizumab 160 mg at week 0; 80 mg every 2 weeks, (10) ustekinumab 45 to 90 mg at weeks 0, 4, 12, (11) Nd: YAG laser, (12) tofacitinib 5 mg twice daily, and (13) tofacitinib 10 mg twice daily, (14) golimumab 2 mg per kg at weeks 0, 4; every 8 weeks, (15) triamcinolone acetonide 2.5 mg per mL, (16) triamcinolone acetonide 5 mg per mL, (17) triamcinolone acetonide 7.5 mg per mL, (18) triamcinolone acetonide 10 mg per mL, (19) methotrexate 25 mg per mL, (20) cyclosporine 50 mg per mL, (21) methotrexate 15 to 20 mg once a week, and (22) cyclosporine 2.5 mg per kg twice a day for 12 weeks; 2.5 to 3.5 mg per kg once daily. The placebo and vehicle arms were amalgamated into one node.

**TABLE 1:** Study‐level characteristics of included studies.

Author	Year	Arm	Route	Dose and frequency	N	Age (±SD)	Sex (percent male)
El‐Basiony	2024	Nd: YAG laser	Topical		43	NR	NR
		Placebo			43		
NCT03991936	2024	Triamcinolone acetonide	Intralesional	2.5 mg per mL	10	NR	NR
				5 mg per mL	10	NR	NR
				7.5 mg per mL	10	NR	NR
				10 mg per mL	10	NR	NR
		Placebo			10	NR	NR
Kristensen	2022	Risankizumab	Subcutaneous	150 mg at weeks 0, 4, 16	72	NR	NR
		Placebo			34		
NCT03611751	2022	Deucravacitinib	Oral	6 mg daily	69	46.9 (13.37)	65.80%
		Placebo			38	47.3 (13.57)	71%
Blauvelt	2021	Ixekizumab	Subcutaneous	160 mg at week 0; 80 mg every 4 weeks	520	49 (13.9)	65%
		Guselkumab	Subcutaneous	100 mg at weeks 0, 4, 12, 20	507	49 (14.88)	61.9%
Mease	2020	Ixekizumab	Subcutaneous	160 mg at week 0; 80 mg every 4 weeks	283	47.5 (12)	57.2%
		Adalimumab	Subcutaneous	40 mg at week 0; 40 mg every other week till week 24	283	48.3 (12.3)	53%
NCT03624127	2020	Deucravacitinib	Oral	6 mg daily	43	45.9 (13.71)	69.30%
		Placebo			34	47.9 (13.98)	68.10%
Mease (NCT02181673)	2020	Golimumab	Intravenous	2 mg per kg at weeks 0, 4; every 8 weeks		NR	NR
		Placebo				NR	NR
Wasel	2020	Ixekizumab	Subcutaneous	160 mg at week 0; 80 mg every 2 weeks	166	43 (12)	36.1%
		Ustekinumab	Subcutaneous	45 mg/90 mg at weeks 0, 4, 12	166	45.4 (12.7)	48.2%
Mease	2019	Etanercept	Subcutaneous	50 mg once a week for 24 weeks	284	48.5 (13.5)	53.2%
		Methotrexate	Subcutaneous	15 to 20 mg once a week	284	48.7 (13.1)	43.7%
Mittal	2018	Triamcinolone acetonide	Intralesional	10 mg per mL	17	NR	NR
		Methotrexate	Intralesional	25 mg per mL	17	NR	NR
		Cyclosporine	Intralesional	50 mg per mL	17	NR	NR
Foley	2018	Guselkumab	Subcutaneous	100 mg at weeks 0, 4, 12, 20	825	43.8 (12.43)	71.4%
		Adalimumab	Subcutaneous	80 mg at week 0; 40 mg every other week till week 24	582	43 (12.29)	72%
Nash	2017	Ixekizumab	Subcutaneous	160 mg at week 0; 80 mg every 4 weeks	122	52.6 (13.6)	51.6%
		Ixekizumab	Subcutaneous	160 mg at week 0; 80 mg every 2 weeks	123	51.7 (11.9)	40.7%
		Placebo			118	51.5 (10.4)	47.5%
NCT01276639	2015	Tofacitinib	Oral	5 mg twice daily	363	45.6 (13.4)	71.9%
		Tofacitinib	Oral	10 mg twice daily	360	45.2 (12.8)	72.5%
		Placebo			177	45 (12.6)	68.37%
NCT01309737	2015	Tofacitinib	Oral	5 mg twice daily	382	45.9 (12.9)	70.16%
		Tofacitinib	Oral	10 mg twice daily	381	44.3 (13.0)	67.5%
		Placebo			196	44.8 (12.6)	62.76%
Kyriakou	2013	Infliximab	Intravenous	5 mg/kg at weeks 0, 2, 6; every 8 weeks till week 24	12	44.92 (15.37)	41.7%
		Adalimumab	Subcutaneous	80 mg at week 0; 40 mg every other week till week 24	14	46.29 (15.68)	35.7%
		Etanercept	Subcutaneous	50 mg twice a week for 12 weeks; 25 mg twice a week for 12 weeks	13	49.15 (12.88)	53.8%
Ortonne	2013	Etanercept	Subcutaneous	50 mg twice a week for 12 weeks; 25 mg twice a week for 12 weeks	36	46.2 (13.5)	72.2%
		Etanercept	Subcutaneous	50 mg once a week for 24 weeks	33	45.4 (9.2)	72.7%
Saraceno	2013	Adalimumab	Subcutaneous	80 mg at week 0; 40 mg every other week till week 24	20	48 (12)	75%
		Etanercept	Subcutaneous	50 mg twice a week for 12 weeks; 25 mg twice a week for 12 weeks	20	45 (10)	70%
		Infliximab	Intravenous	5 mg/kg at weeks 0,2,6; every 8 weeks till week 24	20	50 (15)	70%
Gumusel	2011	Methotrexate	Subcutaneous	15 to 20 mg once a week	17	42.5 (12.5)	58.82%
		Cyclosporine	Oral	2.5 mg per kg twice a day for 12 weeks; 2.5 to 3.5 mg per kg once daily	17	38.4 (10.2)	47.06%

*Note:* The information presented herein is the study‐level (i.e., aggregate‐level) characteristics of the included studies.

Abbreviations: mg, milligram; *N*, sample size at baseline; NR, not reported; SD, standard deviation.

**FIGURE 2:**
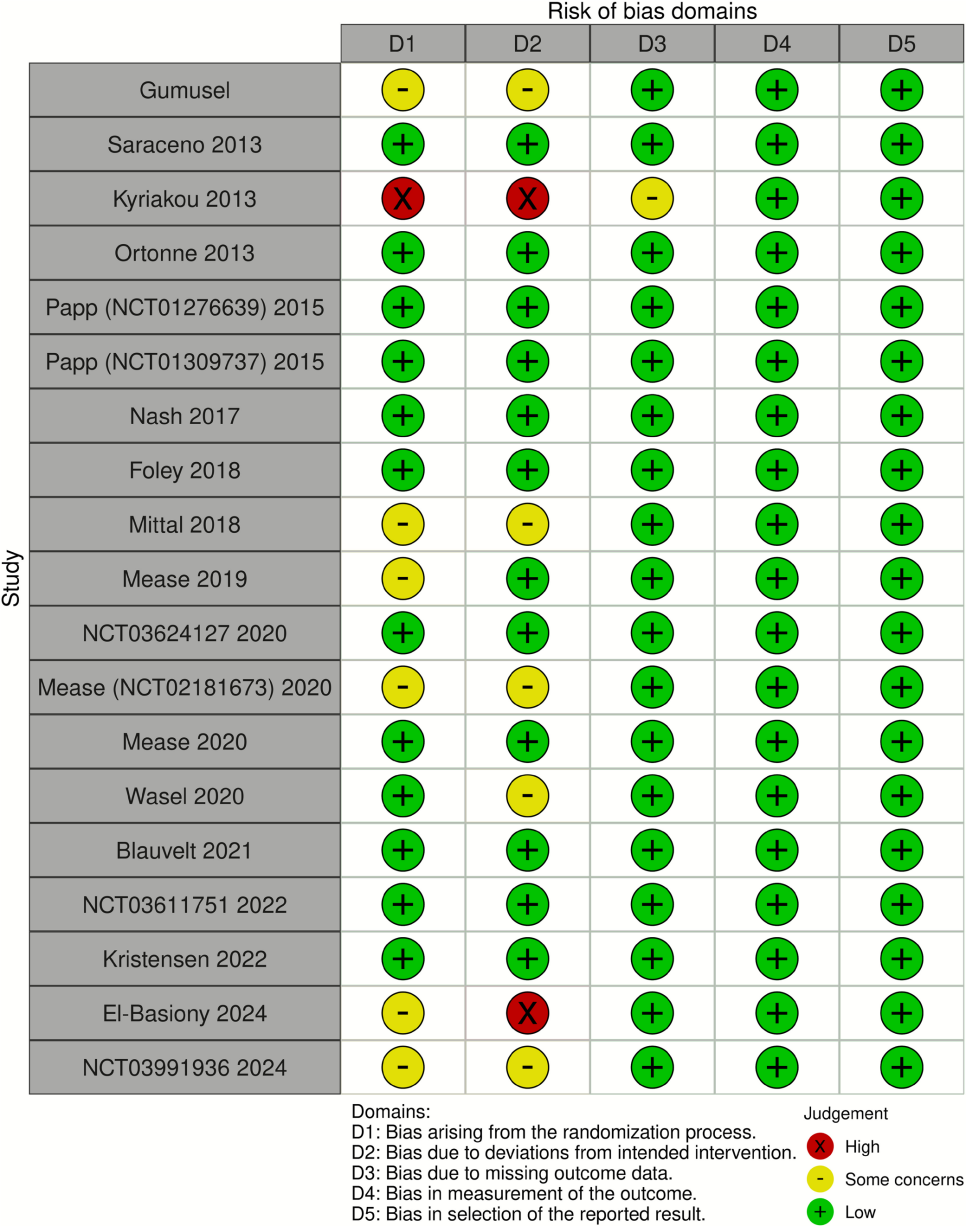
Eligible studies' evidence quality. This “traffic plot” depicts a qualitative evaluation for each study's risk of bias (RoB).

**FIGURE 3:**
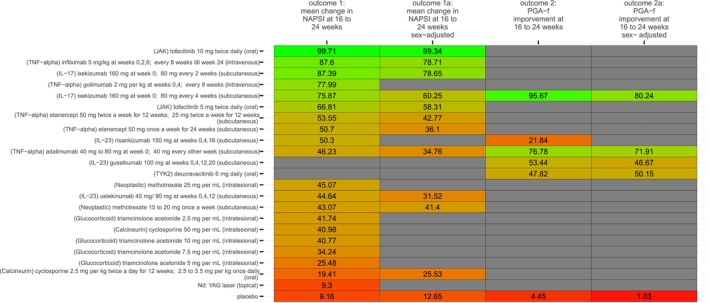
A kilim plot of comparators' surface under the cumulative ranking curve (SUCRA) values. This kilim plot illustrates the relative efficacy of each comparator. The numeric values displayed within each cell correspond to the SUCRA values of the comparators. The horizontal axis represents the outcomes, while the vertical axis corresponds to the individual comparators. The purpose of this kilim plot is to offer an intuitive visual comparison of the relative efficacy of various comparators by way of a color gradient. In this representation, greener cells indicate higher efficacy, whereas redder cells denote lower efficacy. mg, milligrams; NAPSI, Nail Psoriasis Severity Index; PGA‐f, Physician Global; Assessment for fingernails. TNF‐α corresponds to inhibitors of tumor necrosis factor‐alpha: Ustekinumab is also an inhibitor of interleukin 12 (IL‐12); IL‐17 corresponds to interleukin 17 and IL‐23 corresponds to interleukin 23; Calcineurin corresponds to calcineurin inhibitors; JAK corresponds to Janus kinase inhibitors.

**FIGURE 4:**
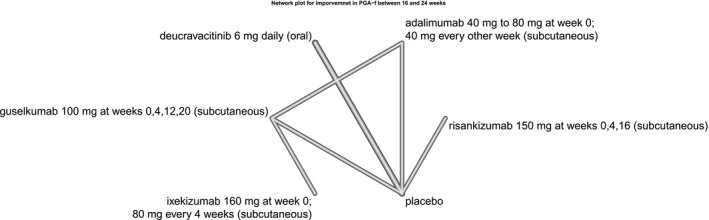
Network plot for PGA‐f. This plot is for the network representing the proportion of patients who achieved a Physician Global Assessment for fingernails (PGA fingernail) of clear or almost clear between 16 and 24 weeks from baseline.

**FIGURE 5:**
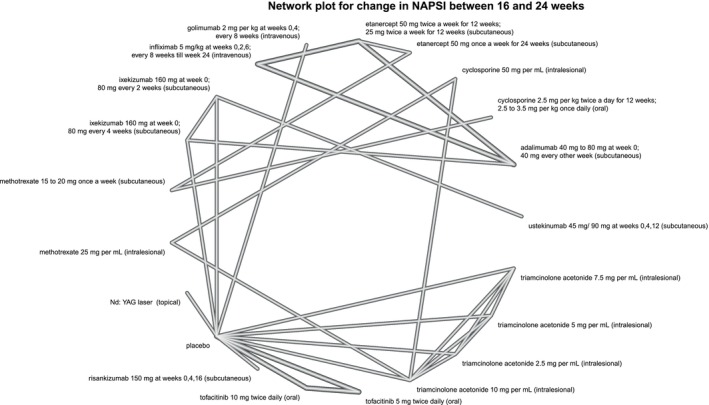
Network plot for NAPSI. This plot is for the network representing the mean change in Nail Psoriasis Severity Index (NAPSI) between 16 and 24 weeks from baseline.

**FIGURE 6:**
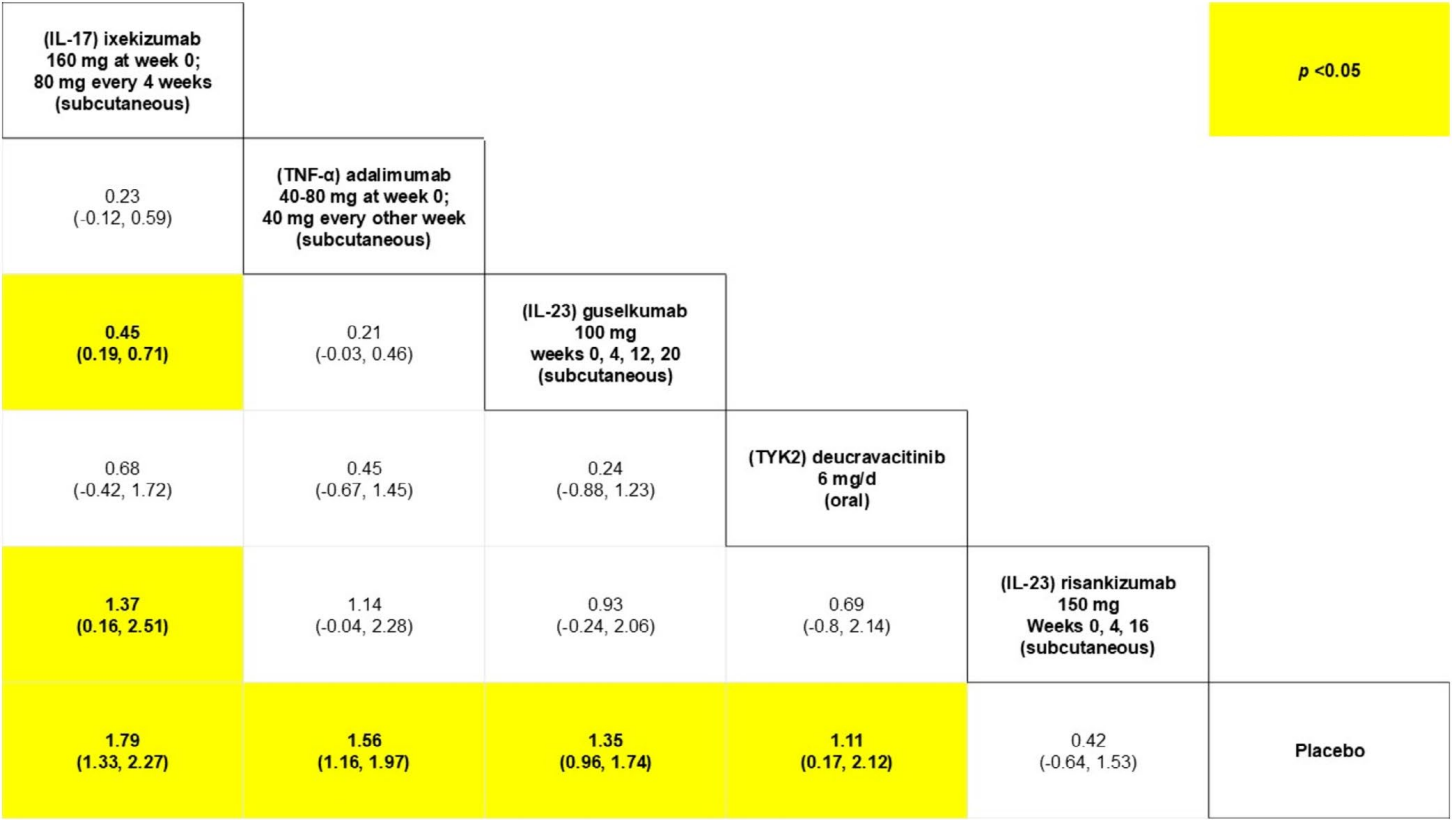
League table for PGA‐f between 16 and 24 weeks from baseline. This table presents comparators' relative efficacy for every possible pairwise comparison. Each cell presents a point estimate (i.e., mean difference of the log odds) and 95% credible interval in parentheses. Yellow‐colored cells represent a statistically significant difference (*p* < 0.05). TNF‐α corresponds to inhibitors of tumor necrosis factor‐alpha; Ustekinumab is also an inhibitor of interleukin 12 (IL‐12); IL‐17 corresponds to the inhibitor of interleukin 17 and IL‐23 corresponds to the inhibitor of interleukin 23.

**FIGURE 7:**
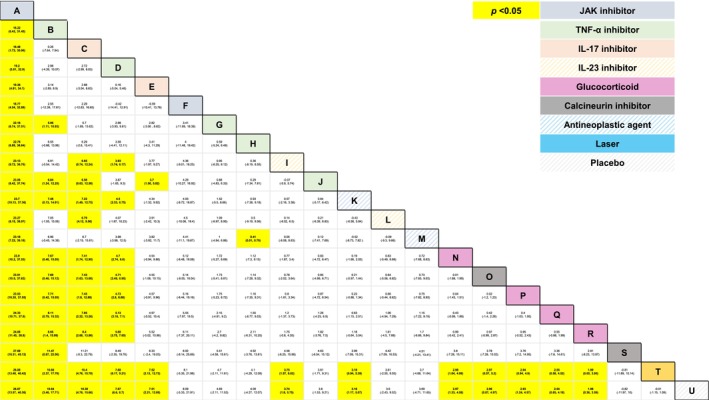
League table for mean change in NAPSI between 16 and 24 weeks from baseline. This table presents comparators' relative efficacy for every possible pairwise comparison. Each cell presents a point estimate (i.e., mean difference) and 95% credible interval in parentheses. Yellow‐colored cells represent a statistically significant difference (*p* < 0.05). A: (JAK) tofacitinib 10 mg twice daily (oral), B: (TNF‐α) infliximab 5 mg/kg at weeks 0, 2, 6; every 8 weeks until week 24 (intravenous), C: (IL‐17) ixekizumab 160 mg at week 0; 80 mg every 2 weeks (subcutaneous), D: (TNF‐α) golimumab 2 mg/kg at weeks 0, 4; every 8 weeks (intravenous), E: (IL‐17) ixekizumab 160 mg at week 0; 80 mg every 4 weeks (subcutaneous), F: (JAK) tofacitinib 5 mg twice daily (oral), G: (TNF‐α) etanercept 50 mg twice a week for 12 weeks; 25 mg twice a week for 12 weeks (subcutaneous), H: (TNF‐α) etanercept 50 mg once a week for 24 weeks (subcutaneous), I: (IL‐23) risankizumab 150 mg at weeks 0, 4, 16 (subcutaneous), J: (TNF‐α) adalimumab 40–80 mg at week 0; 40 mg every other week (subcutaneous), K: (Anti‐neoplastic) methotrexate 25 mg/mL (intralesional), L: (IL‐23) Ustekinumab 45 mg/90 mg at weeks 0, 4, 12 (subcutaneous), M: (Anti‐neoplastic) methotrexate 15–20 mg once a week (subcutaneous), N: (Glucocorticoid) triamcinolone acetonide 2.5 mg/mL (intralesional), O: (Calcineurin) cyclosporine 50 mg/mL (intralesional), P: (Glucocorticoid) triamcinolone acetonide 10 mg/mL (intralesional), Q: (Glucocorticoid) triamcinolone acetonide 7.5 mg/mL (intralesional), R: (Glucocorticoid) triamcinolone acetonide 5 mg/mL (intralesional), S: (Calcineurin) cyclosporine 2.5 mg/kg twice a day for 12 weeks; 2.5–3.5 mg/kg once daily (oral), T: Nd:YAG laser (topical), U: Placebo. TNF‐α corresponds to inhibitors of tumor necrosis factor‐alpha; Ustekinumab is also an inhibitor of interleukin 12 (IL‐12); IL‐17 corresponds to inhibitor of interleukin 17 and IL‐23 corresponds to inhibitor of interleukin 23; Calcineurin corresponds to calcineurin inhibitors; JAK corresponds to Janus Kinase inhibitor; TYK2 corresponds to the inhibitor of Tyrosine Kinase 2.

Qualitative summary of each study's risk of bias is summarized in the traffic plot in Figure 2. The comparators' SUCRA values across each network (i.e., across each outcome) under the base and sex‐adjusted (i.e., sensitivity) analyses are presented in the kilim plot [38] in Figure 3. The network plots for the two outcomes are in Figures 4 and 5; the relative effects are presented in the league tables in Figures 6 and 7. Node splitting analysis of consistency was not possible for either network due to the absence of a closed loop [39].

## 4 Discussion

We determined the relative effects of monotherapy with various agents, including methotrexate, cyclosporine, inhibitors of interleukin 17 (IL‐17), and interleukin 23 (IL‐23), on NP using the current evidence base. Our current NMA study and those by Husein‐El Ahmed et al. [10], Szebényi et al. [12], Huang et al. [13], and Egeberg et al. [11] collectively highlight the salience of having clinical trials primarily designed for the study of NP. The existing comparative data on NP treatments from the current study largely come from trials that were primarily designed for plaque psoriasis, and empirical data regarding NP treatments usually come from post hoc analyses. Hence, selection bias due to post hoc selection [40] cannot be ruled out from our NMA study.

We used well‐recognized outcome measures for the analyses, namely, NAPSI and PGA‐f; these have been used in numerous NMA studies, including the previous NMA studies by Husein‐El Ahmed et al. [10], Szebényi et al. [12], Huang et al. [13], and Egeberg et al. [11].

Our results resonate with those of previous NMA studies. For instance, our analyses ranked “tofacitinib 10 mg twice daily” the most efficacious option in terms of 16 to 24 week mean change in NAPSI. When other outcomes are considered, for example, the proportion of individuals attaining PGA‐f of 0 or 1 at 16 to 24 weeks, “ixekizumab 160 mg at week 0, then 80 mg every 4 weeks” was ranked most efficacious. The rank order of our NMA for ixekizumab and tofacitinib tally with works by Huang et al. [13] (who found tofacitinib to be most efficacious) and Egeberg et al. [11] (who found ixekizumab to be the most efficacious). Like the NMAs by Egeberg et al. [11] and Szebényi et al. [12], we found that ustekinumab was ranked lower than, say, tofacitinib and ixekizumab.

For each of the networks, nodes were defined at the dosage level. Furthermore, we identified regimens that had never been compared in previous NMAs for nail psoriasis including: (1) deucravacitinib 6 mg daily, (2) risankizumab 150 mg at weeks 0, 4, 16, and (3) Nd: YAG laser. The time point we chose for our NMAs (16 to 24 weeks) was different from those selected in some of the other NMAs because most of the studies with the different biologics report their efficacy at this time point, making it useful for comparison purposes; notwithstanding that, the relative longer‐term efficacy of the different biologics may be different from the efficacy at weeks 16–24.

The rank order of treatments under sensitivity analyses was similar to those of the base analyses which thereby supports our base model being robust. For sensitivity analyses, we chose to adjust for variation in sex as the literature reports NP to affect men disproportionately more than women [6, 7].

Our results serve as some form of evidence to guide clinical decision‐making because this condition has lacked sufficient clinical evidence base for establishing clinical practice guidelines specific to NP—as echoed by Crowley et al. [4].

## Author Contributions

Conception of the manuscript was done by A.K.G. and M.T. Data analysis was performed by M.A.B. The manuscript was drafted by A.K.G., T.W., M.T., and M.A.B.; substantively edited and revised by A.K.G., M.A.B., T.W., and M.T.

## Funding

The authors have nothing to report.

## Ethics Statement

The authors have nothing to report.

## Consent

The authors have nothing to report.

## Conflicts of Interest

The authors declare no conflicts of interest.

## Data Availability

The data that support the findings of this study are available from the corresponding author upon reasonable request.
